# A Synthetic Aptamer-Drug Adduct for Targeted Liver Cancer Therapy

**DOI:** 10.1371/journal.pone.0136673

**Published:** 2015-11-02

**Authors:** Thu Le Trinh, Guizhi Zhu, Xilin Xiao, William Puszyk, Kwame Sefah, Qunfeng Wu, Weihong Tan, Chen Liu

**Affiliations:** 1 Department of Pathology, Immunology and Laboratory Medicine, College of Medicine, University of Florida, Gainesville, Florida, United States of America; 2 Departments of Chemistry, and Physiology and Functional Genomics, Center for Research at the Bio/Nano Interface, Shands Cancer Center, UF Genetics Institute and McKnight Brain Institute, University of Florida, Gainesville, Florida, United States of America; 3 Molecular Sciences and Biomedicine Laboratory, State Key Laboratory for Chemo/Biosensing and Chemometrics, College of Biology and College of Chemistry and Chemical Engineering, Hunan University, Changsha, China; SAINT LOUIS UNIVERSITY, UNITED STATES

## Abstract

AS1411 (previously known as AGRO100) is a 26 nucleotide guanine-rich DNA aptamer which forms a guanine quadruplex structure. AS1411 has shown promising utility as a treatment for cancers in Phase I and Phase II clinical trials without causing major side-effects. AS1411 inhibits tumor cell growth by binding to nucleolin which is aberrantly expressed on the cell membrane of many tumors. In this study, we utilized a simple technique to conjugate a widely-used chemotherapeutic agent, doxorubicin (Dox), to AS1411 to form a synthetic Drug-DNA Adduct (DDA), termed as AS1411-Dox. We demonstrate the utility of AS1411-Dox in the treatment of hepatocellular carcinoma (HCC) by evaluating the targeted delivery of Dox to Huh7 cells *in vitro* and in a murine xenograft model of HCC.

## Introduction

To date the only curative treatments for liver cancer are liver transplant or tumor resection. Often liver cancer is detected at late stage and there are currently few chemotherapeutic treatments available. Sorafenib and doxorubicin are drugs which form the basis of systemic chemotherapy treatments used to treat liver cancer. However these treatments are palliative and only delay tumor development and progression. These untargeted treatments of liver cancer are not highly successful, and may cause iatrogenic disease due to off-target side effects. Therefore treatment approaches that specifically target tumor tissue are highly desirable. Targeted therapy achieves selective delivery of therapeutics mediated by recognition elements that can bind with high affinity to overexpressed receptors on target cancer cells [1]. Possible recognition elements include; antibodies [2], vitamins [3,4], and notably aptamers [5]. Nucleic acid aptamers are single-stranded (ss) oligonucleotides with unique intramolecular conformations with the ability to recognize specific molecular targets. Oligonucleotide aptamers are usually isolated through Systematic Evolution of Ligands by Exponential Enrichment (SELEX) [6,7]. Cancer cell-specific aptamers can be isolated directly using living cancer cells, using Cell-SELEX [8]. Aptamers hold many advantages over other recognition elements [9]. SELEX is typically more efficient and cost-effective than antibody development. Advantagous features of oligonucleotide aptamers include; ease of automated synthesis, ease of modification of functional moieties, high stability, long shelf-life, low immunogenicity, and the ease of antidote development by antisense DNA synthesis [10,11]. These advantages make aptamers an attractive approach for biomedical or clinical applications, including targeted therapy.

The conjugates of drugs and recognition elements (e.g. antibody-drug conjugates) have been intensively studied by leading pharmaceutical companies in order to achieve targeted therapy [2,12]. Aptamers, owing to their intrinsic advantages, are expected to play an important role in targeted delivery of therapeutics in the future. The ease of site-specific chemical modification of aptamers greatly facilitates the conjugation with drugs, including chemotherapeutics and photosensitizers, or with nanomaterials, such as carbon nanotubes or gold nanoparticles [13,14]. In addition, nucleic acid aptamers are highly stable, providing valuable opportunities to design aptamer-therapeutic conjugates through a variety of chemical reactions or by the formation of physical complexes. We have developed a synthetic drug-DNA adduct (DDA) technology for easy and efficient drug-DNA conjugation, inspired by the naturally formed adducts between nucleic acids and various chemicals, such as those formed between genomic DNA and Dox during the administration of Dox. DDAs are stable at relatively low temperatures (e.g. 4°C) for long-term storage, but are able to release drugs at physiological temperatures (about 37°C). Remarkably, the DNA backbone of DDAs is also resistant to nuclease cleavage, most likely due to the steric hindrance preventing nuclease binding. The combined features of DDA technology make it a promising approach for the development of novel therapeutics for targeted liver cancer therapy.

In this work, we focused on AS1411 (AGRO100), a well-known DNA aptamer, currently in phase II clinical trials for treatment of acute myeloid leukemia (AML) and renal cell carcinoma (RCC) [15,16]. Multiple studies have shown that AS1411 binds to plasma membrane nucleolin, which is highly expressed by many cancer cell types, and induces tumor cell apoptosis [17–19]. Although the effects of AS1411 have been assessed in many cancers, the therapeutic potency of this aptamer is often limited. To address this issue, here we developed an AS1411-doxorubicin adduct (AS1411-Dox) to combine the targeting ability of AS1411 and the therapeutic potency of Dox. We evaluated the utility of AS1411 and AS1411-Dox in a hepatocellular carcinoma (HCC) model. The results identify the AS1411–Dox adduct as a novel drug for treatment of HCC.

## Materials and Methods

### Ethics statement

Samples were obtained with the approval of the University of Florida Gainesville Health Science Center Institutional Review Board (IRB-01). Paired tumor and non-tumor liver tissues were used in this study. We also obtained separate approval for the generation of the cell line HCO2 (developed from a HCV/HBV-free HCC tissue in our lab). No donor organs were obtained from executed prisoners or other institutionalized persons. Written informed consents were obtained from all subjects. All animal studies were approved by the University of Florida Institutional Animal Care and Use Committee (IACUC).

### Aptamer DNA preparation

All DNA probes were synthesized on an ABI3400 DNA/RNA synthesizer (Applied Biosystems, Foster City, CA, USA). Biotin was coupled on the 5’-end of DNA probes for flow cytometry analysis. The completed sequences were then de-protected in AMA (ammonium hydroxide/ 40% aqueous methylamine 1:1) at 65°C for 30 min and further purified by reverse-phase HPLC (ProStar, Varian, Walnut Creek, CA, USA) on a C-18 column using 0.1 M triethylamine acetate (TEAA, Glen Research Corp.) and acetonitrile (Sigma Aldrich, St. Louis, MO) as the eluent. The DNA products were dried and then detritylated by dissolving and incubating DNA products in 200 μL 80% acetic acid for 20 minutes. The detritylated DNA products were then precipitated with NaCl (3 M, 25 μL) and ethanol (600 μL). UV-Vis measurements were performed with a Cary Bio-100 UV/Vis spectrometer (Varian) for probe quantification.

### Cell culture

All cancer cell lines except HCO2 were obtained from the American Type Culture Collection (Manassas, VA). HCO2 was developed from a HCV/HBV-free HCC tissue in our lab. Hu767, a primary hepatocyte sample, was obtained from a healthy donor from Cellz Direct (Raleigh, NC). Cells were cultured in DMEM medium supplemented with 10% fetal bovine serum (FBS) (heat-inactivated, GIBCO) and 100 IU/mL penicillin-streptomycin (Cellgro) at 37°C in a humid atmosphere with 5% CO_2_. The cell density was determined prior to each experiment using a hemocytometer.

### Preparation and characterization of AS1411-Dox adduct

Using a modified protocol [20], AS1411-Dox adduct was prepared by incubating Dox (500 μM), DNA (20 μM), and Formaldehyde (0.37%) in reaction buffer (20 mM sodium phosphate, 150 mM NaCl, and 0.5 mM EDTA; pH 7.0) at 10°C overnight. The resultant DDA was purified by reverse phase high-performance liquid chromatography (HPLC) (ProStar, Varian, Walnut Creek, CA, USA) on a C-18 column, using 0.1 M trithylamine acetate (TEAA, Glen Research Corp.) and acetonitrile (Sigma Aldrich, St. Louis, MO) as the eluent. Purified samples were lyophilized, desalted, and dissoved in Dulbecco’s buffer (Sigma Aldrich), and stored at -20°C for future use. Molar extinction coefficients of 11,500 M^-1^cm^-1^ at 480 nm for free Dox, and 7,677 M^-1^cm^-1^ at 506 nm for covalently bound Dox in adduct were used for drug quantification by UV-Vis spectrometry [20].

### Study of specific binding ability and binding affinity

The binding abilities of aptamers or drug-aptamer adducts (final DNA concentrations: 200 nM) were determined using flow cytometry. Cells (2×10^5^) were incubated in binding buffer (200 μL, 4.5 g/L glucose, 5 mM MgCl_2_, 0.1 mg/mL yeast tRNA (Sigma Aldrich) and 1 mg/mL BSA (Fisher Scientific) in Dulbecco’s PBS (Sigma)) on ice for 30 min, followed by washing twice with washing buffer (1 mL, 4.5 g/L glucose and 5 mM MgCl_2_ in Dulbecco’s PBS (Sigma)). Precipitated cells were suspended in binding buffer (200 μL) prior to flow cytometry analysis on a FACScan cytometer (BD Immunocytometry Systems). Data were analyzed with the WinMDI software. The binding affinity of each probe was determined using a series of probe concentrations. As negative controls, similar assays were performed using random DNA sequences (random library) at the same corresponding concentrations. The increased mean fluorescence intensity of cells bound by dye-labelled probes was compared with cells bound by dye labelled random sequences. These fluorescence values were used to calculate the equilibrium dissociation constant (*K*_*d*_) by fitting the dependence of fluorescence intensity of cells bound by probes (*F*) on probe concentration (*L*) to equation:

F=Bmax[L]/(Kd+[L])

Where B_max_ represents the maximum binding capacity. Each binding assay experiment was repeated independently in triplicate.

### Intracellular drug release

Drug release in cells was evaluated using confocal laser scanning microscopy (CLSM). All cellular fluorescent images were collected on a Leica TCS SP5 confocal microscope (Leica Microsystems Inc., Exton, PA) with a 63x oil immersion objective and Leica Confocal Software. Cells were observed in DIC mode. Cells were treated with free Dox (2 μM), AS1411-Dox adduct, or control DNA-Dox adduct (2 μM Dox equivalents). Drugs were suspended in Dulbecco’s PBS and cells were incubated (37°C, 5% CO_2_) for 1 h. Incubation was followed by Hoechst staining with Hoechst 33342 (10 μg/mL) (Molecular Probes, Inc., Eugene, OR) for 30 min before washing with Dulbecco’s PBS. The resultant cells were then observed under microscope.

### Selective cytotoxicity

Cell cytotoxicity was evaluated using CellTiter 96 cell proliferation assay (Promega, Madison, WI, USA). Cells (5 × 10^4^ /well) were treated with free Dox, AS1411, control DNA-Dox adduct, and AS1411-Dox adduct (respectively) in FBS-free medium. All treatments were suspended in PBS. After incubation for 1 h (37°C, 5% CO_2_), medium was removed, and fresh medium (10% FBS, 100 IU/mL penicillin-streptomycin, 200 μL) was added for further cell growth (48 h). Then medium was removed again, and CellTiter reagent (20 μL) diluted in fresh medium (100 μL) was added to each well and incubated for 1–2 h. The absorbance (490 nm) was recorded using a microplate reader (Tecan Safire microplate reader, AG, Switzerland). Cell viability was determined according to the manufacturer’s description.

### Immunostaining of tissues using AS1411-Biotin

FFPE (formalin fixed paraffin embedded) liver tumor tissue sections obtained from patients were subjected to immunostaining using AS1411 aptamer. The tissue sections were deparaffinized twice in xylene for 15 min each, washed with series of 100%, 95%, 80% and 70% for 1 min each and rinsed with distilled water. Tissue sections were then incubated in 10 mM sodium citrate buffer (pH 6.2) at 95°C for 30 min, samples were then microwaved for 2 min and allowed to cool at room temperature. After being washed twice in PBS for 5 min each, tissue sections were incubated in 3% H_2_O_2_ in PBS for 30 min then blocked with biotin-blocking solution (Vector, Laboratories). Tissue sections were then probed with 200 nM AS1411-Biotin for 1h at room temperature and washed 3 times in 0.5% PBST and incubated with HRP-conjugated streptavidin solution (Dako) for 30 min at room temperature. Finally, tissue sections were treated with DAB peroxidase substrate solution (Dako) until color developed (approximately 5 min). Stained tissue sections were examined and pictures were taken by a light microscope.

### Immunocytochemistry of liver cancer cells using AS1411-FITC

Cells were seeded on Superfrost Microscope Slide (Fisherbrand) on the day before staining. Cell slides were washed twice in PBS for 5 min each, blocked in 5% BSA in PBS and incubated with 200 nM of AS1411-FITC for 1h at room temperature. Slides were then washed 3 times in 0.5% PBST and mounted with Vectashield mounting reagent containing DAPI (Vector Laboratories). Slides were examined under a confocal microscope.

### Western blotting analysis

Heart, kidney and tumor tissues were collected from each mouse at the termination of the study. Tissues were lysed in RIPA buffer containing Proteinase Inhibitor Cocktail (Sigma Aldrich) and incubated on ice for 30 min. After centrifugation at 13,300 x g for 15 min at 4°C, the supernatant was transferred to a fresh tube and the concentration of the protein was determined by Bio-Rad Protein Assay (Bio-Rad) according to the manufacturer’s protocol. Protein was analyzed on 12% SDS-PAGE gels. The proteins were then transferred onto nitrocellulose membranes and probed with cleaved caspase-3 antibody (Cell Signaling Technology, Inc.), followed by the appropriate secondary antibody with HRP conjugated (Santa Cruz Biotechnology, Inc.). Immunoreactive bands were detected using SuperSignal West Pico Substrate (Thermo Scientific).

#### Cell cycle detection by Flow cytometry using Propidium Iodide

Each well in a 6-well plate was seeded with 100,000 Huh7 liver cancer cells and treated with 5 μM of AS1411 for 24h or 48h. At the end of the treatment, cells were trypsinized and then collected and washed twice in ice cold PBS. Cells were then fixed with ice cold 70% ethanol at -20°C for 30 min. Cells were then washed twice in cold PBS and subsequently incubated with freshly made 20 μg/ml propidium iodide and 100 μg/ml RNaseA in cold PBS for 1h at 4°C in the dark. The cell cycle was analyzed by flow cytometry followed by the application of FlowJo software.

### In vivo experiments

NOD. Cg-Prkdc (scid) IL2 mice were purchased from Jackson Laboratory (Bar Harbor, ME) and housed at the animal facility of the University of Florida, the protocol was approved by Institutional Animal Care and Use Committee. The tumor xenograft mouse model was developed by subcutaneously injecting 5x10^6^ of in vitro-propagated Huh7 HCC cells (in 100 μL DPBS buffer) into mice on the right flank. Dorsal tumor nodules were allowed to grow to a volume of ~ 100 mm^3^ before treatment initiation. Tumor-bearing mice were randomly assigned to four groups, with 5 mice in each group: (i): treated with AS1411; (ii): treated with free Dox, (iii): treated with control DNA-Dox adduct; and (iv): treated with AS1411- Dox adduct. The doxorubicin dosage was kept the same in groups (ii), (iii) and (iv) at 2 mg/kg; and the AS1411 dosage in group (i) was accordingly maintained as equal to that in group (iv). Drugs were administered through tail vein injection every other day followed by tumor size measurement. The body weight of each mouse was also measured every other day to monitor potential drug toxicity. At the end of the study the mice were humanely sacrificed; heart, kidney and tumor tissues were collected when tumor volume exceeded 2000 mm^3^ or developed ulceration.

## Results and Discussion

### AS1411 Aptamer binding affinity for liver cancer cell lines and patient tumor tissue

**Fig 1:**
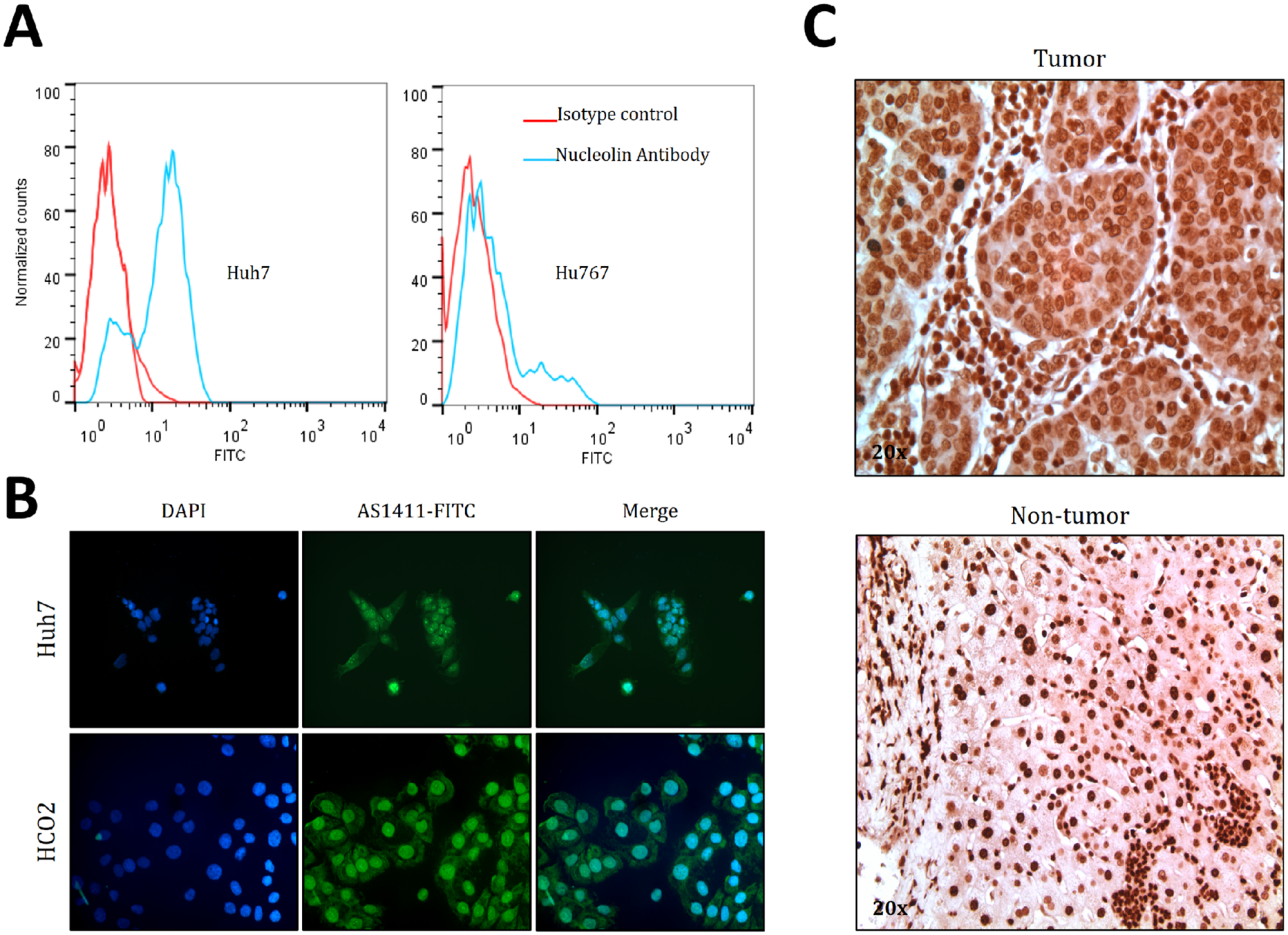
Binding affinity of AS1411 towards HCC cell lines and tissues. **(a)** Nucleolin is detected on cell surface of Huh7 (left) but not on Hu767 (primary hepatocyte, right). 1 million cells of each cell line were incubated with 2ug/ml Nucleolin Antibody and 1ug/ml secondary antibody of mouse-FITC for 1h at 4°C, which was followed by flow cytometry. **(b)** AS1411 can stain membrane nucleolin on Huh7 and HCO2. Live cells were incubated with 200 nM of AS1411-FITC for 30 min at room temperature and examined under a confocal microscope. **(c)** AS1411 stains membrane nucleolin in tumor tissues, but only nuclear nucleolin of non-tumor tissue. Paraffin embedded tissue samples from patients were incubated with 200 nM of AS1411-Biotin for 1 h at room temperature. Signal was developed using HRP-conjugated streptavidin and DAB peroxidase substrate (Dako).

Nucleolin is aberrantly expressed on the cell membrane of many tumor types including glioma, prostate and liver cancer [21–24]. As AS1411 works by first binding to cell membrane nucleolin [19], we sought to determine the presence of cell-surface nucleolin expression in the HCC cell line Huh7 and in a culture of human primary hepatocytes Hu767. By flow cytometry analysis of immunostained cells, we observed that nucleolin was expressed on the cell membrane of Huh7 cells but not in Hu767 cells (Fig 1A). To determine whether AS1411 could be useful as a marker for HCC tumor imaging, we conjugated AS1411 to FITC and performed staining on two HCC cell lines; Huh7 cells and HCO2 cells, a novel HCC cell line developed in our lab (Fig 1B). Likewise, AS1411-Biotin was used to develop immunohistochemistry staining of paraffin embedded HCC patients tumor samples. 21 HCC tumor tissue samples (formalin fixed and paraffin embedded) from HCC patients were analyzed, and in 15 samples AS1411 were found to preferentially bind in tumor regions compared with adjacent non-tumor tissue. AS1411 showed the strongest staining around nucleus, where nucleolin is the most abundant. However, AS1411 also stained the membrane and the cytoplasm of liver cancer cells clearly distinguishing tumor tissue, indicating aberrant transport of nucleolin throughout HCC tumor cells (Fig 1C left panel). Signal detection on non-tumor tissue was confined to the nucleus (Fig 1C right panel). Additional data confirm that AS1411 stains the membrane of tumor cells and preferentially stains liver tumor tissue, compared with the nucleolin antibody which often only stains the nuclear nucleolin (S1 and S2 Figs). The results indicate that AS1411 binds preferentially to liver cancer cells and can distinguish tumor and non-tumor tissue by the difference in staining intensity. The data show that AS1411 is a suitable candidate for further development as a Drug-DNA adduct for the specific targeting of liver cancer.

### Assessment of AS1411 cytotoxicity in HCC cell lines

**Fig 2:**
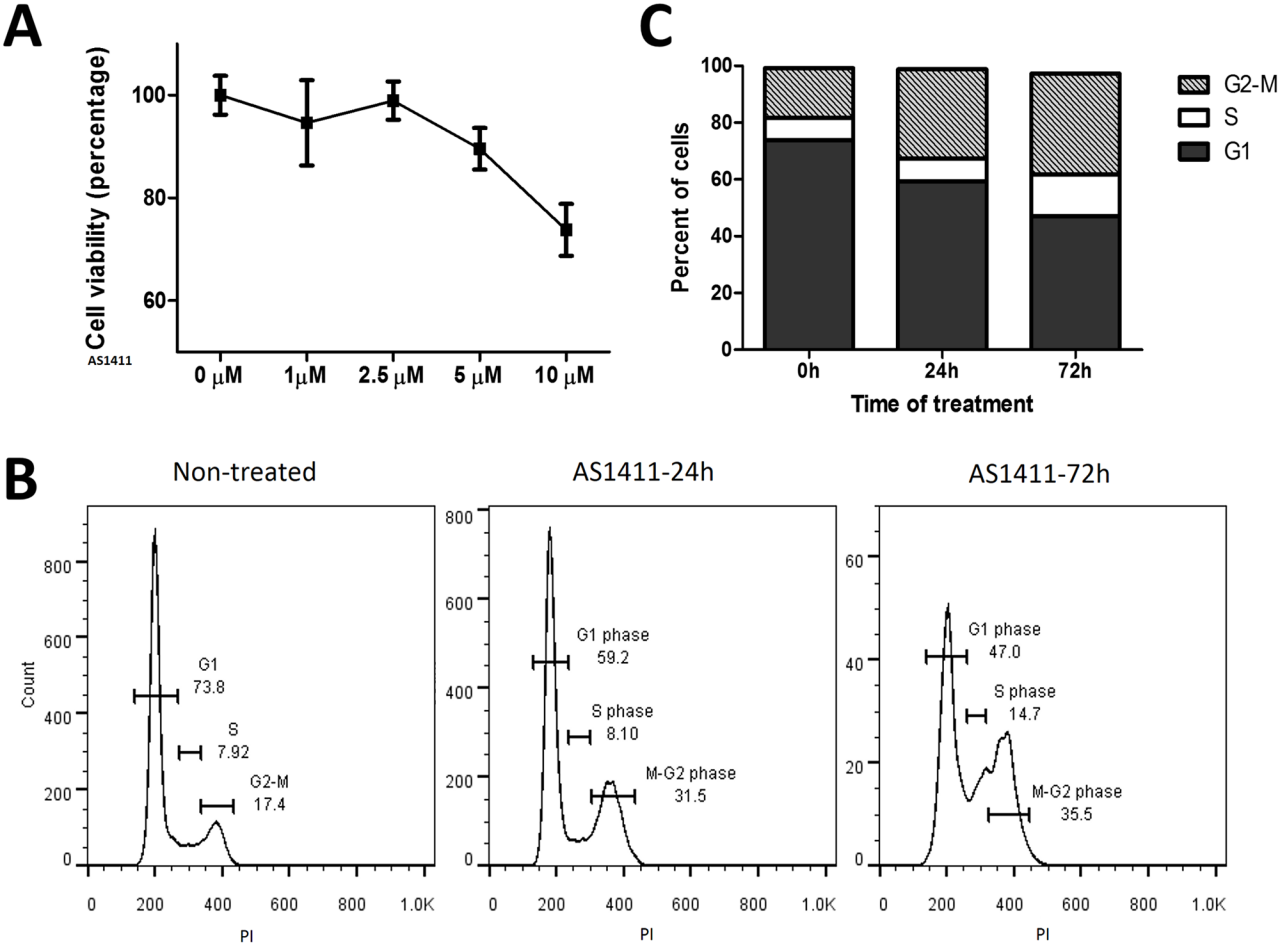
Growth inhibition of Huh7 by treatment with AS1411. **(a)** Huh7 was incubated with AS1411 at concentrations from 1 μM to 10 μM for 2 days at 37°C then subjected to MTS assay. **(b)** Cell cycle arrest was tested by flow cytometry of PI-stained cells. FLOW data indicates cell cycle arrest in AS1411 treated cells and cell cycle percentage **(c)** Analysis of the flow cytometry data showed that a much higher proportion of treated cells were arrested at S and G2/M phases, when compared to untreated cells.

AS1411 has cytotoxic properties against breast cancer and enhances the efficacy of chemotherapy in glioma treatment [25,26]. In order to assess any potential cytotoxic effects of AS1411 on liver cancer cells *in vitro* we incubated Huh7 cells with different concentrations of AS1411 ranging from 1 μM to 10 μM for 48 hours. After incubation cell viability was assessed by MTS assay. No effect was observed in Huh7 cells treated with a low dosage of AS1411. However, decreased cell proliferation was observed in cells treated with AS1411 concentrations of 5 μM or greater (Fig 2A). We found no obvious induction of cell apoptosis, AS1411 was previously reported to induce tumor cell apoptosis [25]. Therefore we sought to examine the extent by which Huh7 cells are inhibited by AS1411. Cell cycle analysis was performed by flow cytometry on cells treated with AS1411 for 24 and 72 hours. Treatment of Huh7 cells with 5 μM AS1411 induced an increase in cells arrested at S phase and G2/M phase. The percentage of cells arrested in S phase increased from 7.92% to 14.7% after 72 hours of treatment (Fig 2B and 2C). A bigger effect was observed upon treatment with AS1411 with cells arrested at G2/M phase; with the percentage of cells arrested increasing from 17.4% to 31.5% after 24 hours of treatment and upto 35.5% after 72 hours of treatment (Fig 2C). Arrest at the G2/M checkpoint may enable cells to undergo apoptosis and efforts to increase G2/M arrest may increase the sensitivity of cells, to chemotherapeutic interventions [27–29]. These results suggest that AS1411 by itself inhibits Huh7 cell proliferation in a dose-dependent manner, and that while AS1411 does not directly induce cytotoxic effects in target Huh7 cells it does inhibit tumor cell proliferation to some extent by inducing G2/M arrest. Although only just over a third of the cells were arrested at G2/M by treatment with 5 μM AS1411, higher dosages may prove more effective, as is indicated by a further reduction of viable cells upon treatment with 10 μM as indicated by the cell viability assay (Fig 2A). This effect may be further enhanced by combination with a chemotherapeutic agent for the treatment of liver cancer. We decided to combine doxorubicin (Dox) with AS1411 to form the AS1411-Dox adduct.

### Preparation and utility of the AS1411-Dox adduct

**Fig 3:**
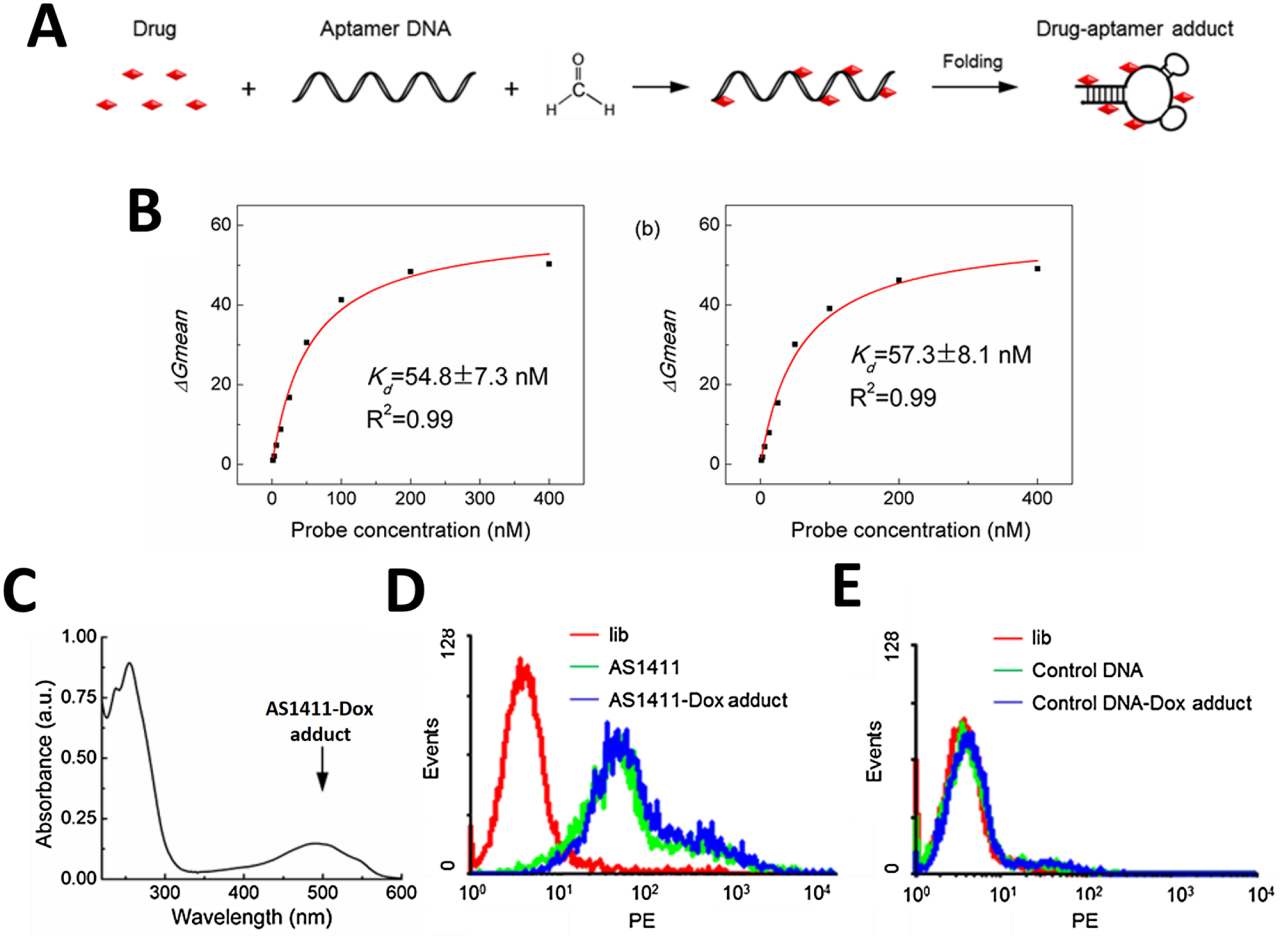
Preparation and affinity of the AS1411-Dox adduct. **(a)** Schematic description of the preparation of AS1411-Dox adduct by incubating Dox (500 μM), AS1411 (20 μM), and formaldehyde (0.37%) at 10°C overnight, followed by purification by reverse phase HPLC. **(b)** AS1411-Dox adduct remained its binding affinity towards Huh7 established by the equilibrium dissociation constant (*Kd*). **(c)** UV-Vis spectrum displaying the absorbance of purified AS1411-Dox adduct, with the characteristic absorbance peak of Dox at 490 nm. **(d)** Flow cytometry results indicating specific recognition ability of AS1411 and AS1411-Dox adduct to Huh7 HCC cells, note the non-specific DNA library does not detect Huh7 cells **(e)** Random library and control DNA aptamers and the control DNA-Dox adduct in contrast were not able to specifically bind to Huh7 cells.

Dox is a widely prescribed chemotherapeutic used in cancer therapy. Dox is capable of forming adducts with DNA [30,31]. The Dox adduct was prepared by incubating Dox with formaldehyde, which was used as a reducing and crosslinking agent during adduct formation. Specifically, AS1411-Dox adduct was prepared by incubating aptamers with Dox and formaldehyde in reaction buffer at 10°C overnight (Fig 3A). Residual aptamer, drug, and formaldehyde were removed from the reaction using reverse phase high-performance liquid chromatography (HPLC). Results indicate that the DDA displayed strong absorbance at 260 nm (from drug and DNA) as well as some absorbance from 490 nm (exclusively from drug) (S3 Fig). Purified adduct was lyophilized and desalted. UV-Vis spectrometry results further verified the characteristic drug absorbance around 490 nm in purified DDA (Fig 3C). Based on previous reports, molar extinction coefficient of 7,677 M^-1^cm^-1^ at 506 nm for Dox in DDA was used for drug quantification in adduct [20]. Accordingly, AS1411-Dox adduct was determined to have 5.4 ± 0.1 (s.d., n = 3) copies of Dox moieties per AS1411 strand. Similarly, a non-binding control DNA (S1 Table) was also used to prepare a control adduct with Dox (control-Dox).

As we have demonstrated, AS1411 was able to bind to target Huh7 liver cancer cells. We then evaluated whether the resultant AS1411-Dox adduct maintained the specific recognition ability to target cancer cells. AS1411 and the control sequence were biotinylated at the 5’ end and used to prepare adducts with Dox. Streptavidin-PE (Phycoerythrin) conjugate dye was used in flow cytometry to monitor the fluorescence of cells incubated with either AS1411-Dox or control-Dox. Unconjugated AS1411 was used as a positive control, and a library of random DNA sequences was used as a negative control. Intense cell staining of Huh7 cells was observed on incubation with the AS1411-Dox adduct, indicating that the drug-aptamer conjugate maintained the specific binding capability of the original aptamer. The fluorescent intensity was comparable to cells incubated with AS1411, suggesting a minimal effect on the binding capability of the adduct, when compared with unconjugated aptamers. In contrast, neither the negative control DNA, nor the control-Dox adduct bound to Huh7 cells (Fig 3D). The binding affinities of AS1411 and the AS1411-Dox adduct for Huh7 cells were further examined by determining the dissociation constant (*K*_*d*_). The AS1411-Dox adduct had a *K*_*d*_ of 57.3 ± 8.1 nM (s.d. n = 3), with AS1411-Dox adduct also binding to target Huh7 cells with strong affinity. The *K*_*d*_ value of AS1411-Dox is comparable to that ofAS1411 (*K*_*d*_ = 54.8 ± 7.3 nM) (Fig 3B). The slightly reduced binding affinity of the AS1411-Dox adduct may be due to slight conformational changes caused by Dox-DNA adduct formation. Dox forms methylene links to N2 of DNA strands (S4 Fig). This is an important issue that requires further study by NMR spectroscopy to elucidate the full structure of AS1411-Dox. However, these effects are minimal and AS1411-Dox retains the ability to specifically bind Huh7 cells. These data provide the basis for downstream applications of targeted liver cancer therapy.

### Intracellular drug release from AS1411-Dox adducts

**Fig 4:**
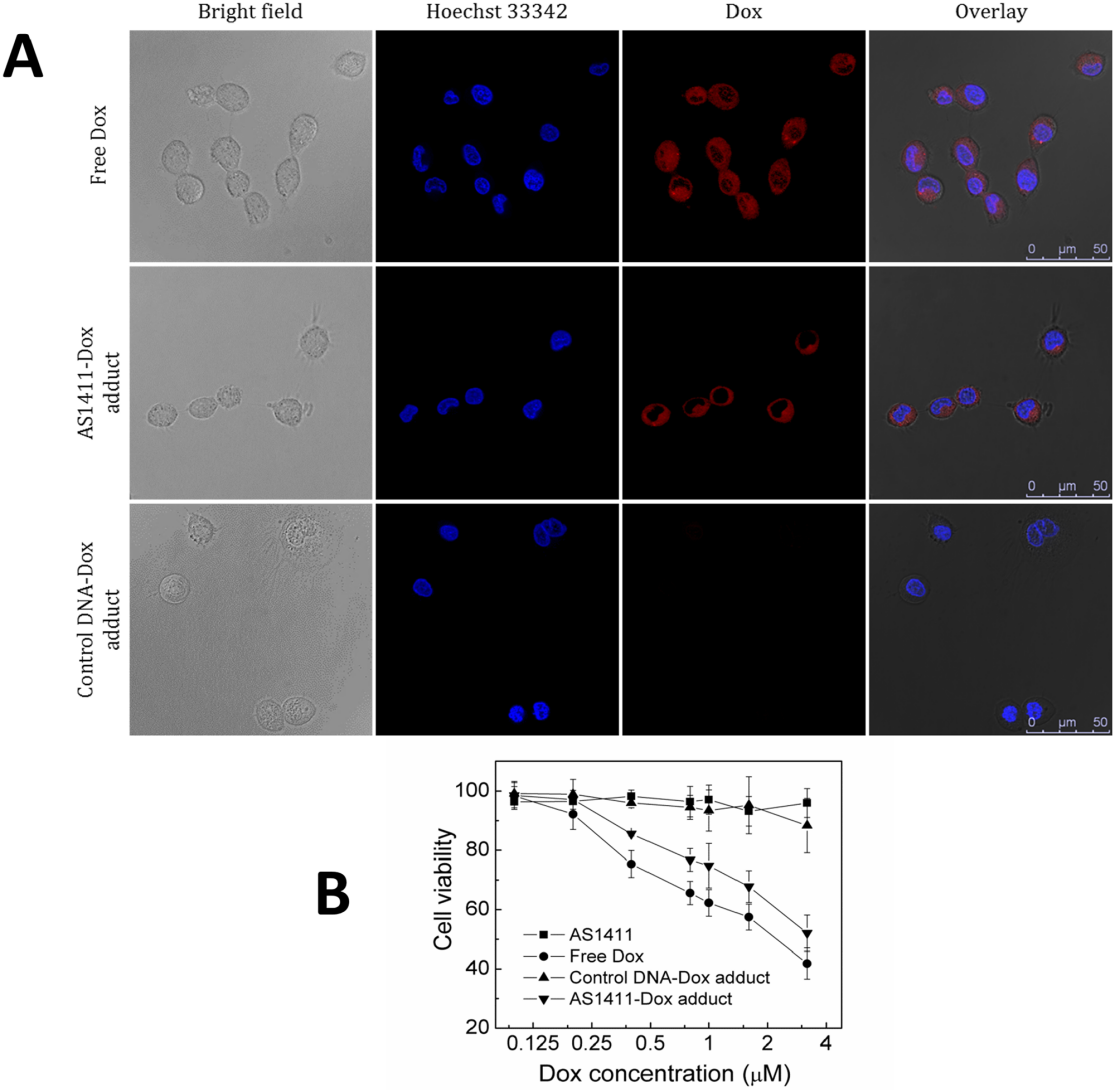
Intracellular staining of free Dox and AS1411-Dox with Control DNA-Dox adduct. **(a)** Confocal laser scanning microscopy images displaying uptake of Dox into Huh7 cells treated with 2 μM of free Dox, AS1411-Dox adduct, or control DNA-Dox adduct, respectively. After treatment cells were stained with Hoechst 33342 (scale bar: 50 μm). Free Dox passed into any cells; however only Dox attached to AS1411 and not the control aptamer was selectively delivered to Huh7 cells due to the binding affinity of AS1411. **(b)** Cells were treated for 1h with either; 2 μM free Dox, AS1411, AS1411-Dox adduct or control-DNA adduct., prior to MTS assay. The results indicate the specific and potent cytotoxicity in target Huh7 cells induced by AS1411-Dox adduct.

The intracellular behaviors of free Dox and the AS1411-Dox adduct were examined using confocal laser scanning microscopy. In this study, Huh7 cells were incubated with either; free Dox, the AS1411-Dox adduct, or the control DNA-Dox adduct. Incubation was followed by staining with Hoechst 33342 to localize nucleus prior to microscopy observation. Free Dox rapidly accumulated in cells, as shown by intense intracellular Dox fluorescence (Fig 4A). Intense drug fluorescence was also observed in Huh7 cells incubated with AS1411-Dox adduct, indicating the uptake of this adduct and drug release from adduct. In contrast, cells treated with control DNA-Dox adduct displayed fairly weak drug fluorescence. Having established that AS1411-Dox adduct can specifically recognize target Huh7 liver cancer cells, and deliver Dox into this target cell, the consequent *in vitro* cytotoxicity was evaluated by an MTS assay. Cells were treated with free Dox and AS1411-Dox adduct, respectively, with a range of doxorubicin concentrations. While neither aptamer AS1411 alone nor control-Dox adduct induced potent cytotoxicity, both free Dox and the AS1411-Dox adduct showed comparable dose-dependent cytotoxicity in target Huh7 cells (Fig 4B). These data show that the AS1411-Dox adducts retain the specificity for binding to Huh7 cells *in vitro* and that the conjugation of Dox to AS1411 does not prevent Dox-mediated cytotoxicity to target cells. Taken together, these data show that the AS1411-Dox adduct is able to specifically target Huh7 cells *in vitro*, efficiently deliver Dox into targets cells and reduce cell viability to a comparable level to that of free Dox. The data also indicate that the adduct formation does not inhibit intracellular release of Dox. These results motivated us to test the efficacy and potential side effects of AS1411-Dox *in vivo*, using a xenograft model of Huh7 tumors in mice.

### In vivo evaluation of AS1411-Dox adduct for targeted liver cancer therapy

**Fig 5:**
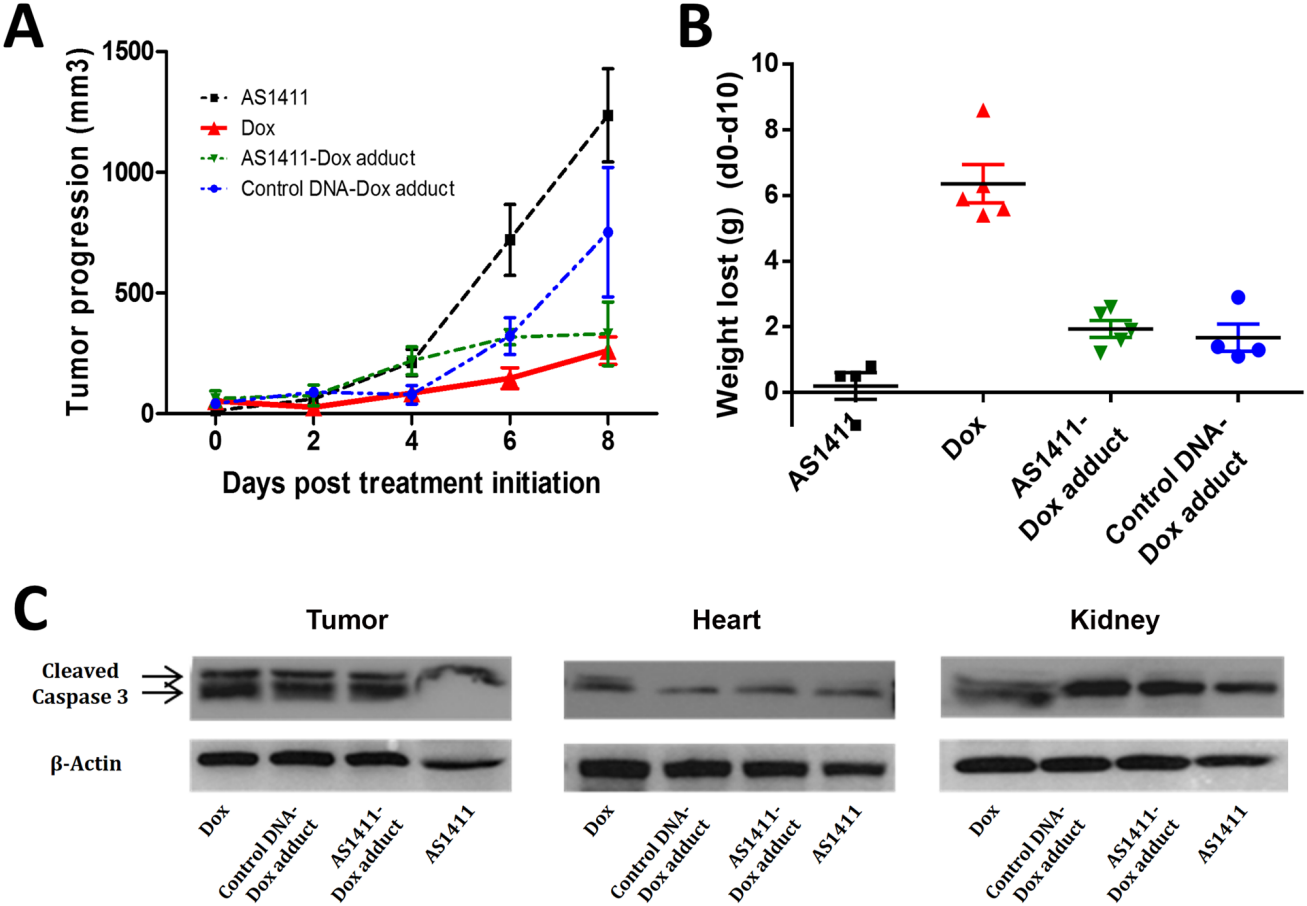
In vivo evaluation of AS1411-Dox adduct in Huh7 tumor xenograft mouse model. **(a)** Mice treated with free Dox or AS1411-Dox adduct had significant lower rates of tumor growth than those treated with AS1411 or control DNA-Dox adduct. **(b, c)** Side effects and cytotoxicity of each drug was illustrated by total body weight lost at the end of treatment (b) or activation of Caspase-3 in tumors, heart and kidney tissues (c). Mice treated with Dox lost 21.7% of their total body weight while with AS1411-Dox only 6.6% (P<0.001). Cleaved Caspase-3, which indicates the activation of Caspase-3 and induced apoptosis, was only identified in the heart and kidney of free Dox-treated mice, suggesting the specific cytotoxicity and reduced side effects of AS1411-Dox adduct compared to Dox.

There are several well documented major side effects of doxorubicin treatment including; weight loss, cardio cytotoxicity and nephro cytotoxicity [32–38]. Doxorubicin is highly toxic and is administered intra-hepatically in normal circumstances [39]. To determine the efficacy of the AS1411-Dox adduct, a murine model of HCC was tested. The AS1411-Dox adduct was prepared as described and determined to have about 5.4 ± 0.1 copies of Dox per each strand of AS1411. Again we used a control DNA sequence which does not bind to target liver cancer cells, to prepare a control-Dox adduct. In brief, NOD Cg-Prkdc (scid) IL2 mice were each inoculated with 1 million Huh7 cells. Dorsal tumor nodules were allowed to grow to a volume of ~100 mm^3^ before treatment initiation. Tumor-bearing mice were randomly assigned into 4 groups (n = 5), and were treated by tail vein injection with either; (i) AS1411, (ii) free Dox, (iii) control-Dox adduct, or (iv) AS1411-Dox adduct, respectively. The Dox dosage was kept at 2 mg/kg, and the dosage of AS1411 in groups (i) and (iv) were equal. Tumor size and mouse weight were measured every other day, as indices of drug efficacy and non-specific cytotoxicity. The average tumor growth rates were calculated accordingly (Fig 5A). The mice treated with free Dox or AS1411-Dox adduct all had significantly lower rates of tumor growth than those treated with AS1411 alone or the control-Dox adduct. The control-Dox adduct also partially inhibited tumor progression, which is somewhat surprising. This is most likely due to the gradual release of Dox from the DNA, during an extended period of time in the circulation, as the control sequence does not target either tumor or normal cells. The antitumor efficacy of AS1411-Dox adduct was less than that of free doxorubicin, however the side effects of AS1411-Dox are greatly reduced compared to free Dox. At the end of treatment, the group of mice treated with free Dox lost 22.4% of total body weight on the average, while the group treated with the AS1411 Dox-adduct lost on average only 7.17% of total body weight (P<0.01) (Fig 5B). These side effects were further studied by examination of induced apoptosis in the heart and kidney tissue of mice treated in groups (i-iv). The induction of apoptosis in tumor tissue and heart and kidney tissue collected from mice was assessed by measuring the presence of cleaved Caspase 3 by western blot analysis. The data show that cleaved Caspase-3 was present in tumor samples collected from mice treated with the AS1411-Dox adduct, control-Dox adduct- and free Dox, indicating the cytotoxicity of Dox towards tumor tissue. There were no detectable levels of cleaved Caspase-3 in tumor samples treated with AS1411 only (Fig 5C). The data suggest that, under our experimental conditions, the aptamer alone did not induce apoptosis or cause cytotoxicity to either tumors or normal healthy tissue and that the effects of conjugating doxorubicin to AS1411 are beneficial. These data corroborate the previous observation that the AS1411 aptamer is not cytotoxic to Huh7 cells but a potent cell cycle inhibitor (Fig 2C). Unsurprisingly treatment with free Dox not only inhibited tumor growth, but also had the side effects of inducing apoptosis in heart and kidney tissues, providing evidence of typical Dox non-specific cytotoxicity, which is well documented. On the other hand, the AS1411-Dox adduct targeted tumor tissue with high specificity and induced tumor cell death, inhibiting tumor growth without inducing apoptosis in non-tumor tissues (Fig 5C). These results imply that the slight reduction in efficacy of AS1411-Dox could be compensated for by an increase in dosage as there were no major side effects detected for this adduct. The data provide evidence to suggest that the AS1411-Dox adduct has a similar tumor inhibition effect as that of free Dox, but with more targeted specificity to tumor and reduced non-specific systemic cytotoxicity to normal tissues. Conjugating Dox to AS1411 enables specific targeting of liver cells, and the majority of Dox is released in tumor cells, which provides a protective effect and prevents non-specific systemic cytotoxicity. The AS1411 aptamer has previously been shown to induce tumor cell apoptosis in glioma, leukemia, breast cancer and renal cell carcinoma [16,18,19,25,26,32]. Two recent studies have looked into the potential use of AS1411 in liver cancer cells. Yu and colleagues assessed the intracellular uptake of AS1411 conjugated to poly lactide co-glycolide (PLGA) nanoparticles [40]. The main findings were that AS1411 coated PLGA particles were preferentially absorbed by the HCC cell line, QGY-7703, when compared with an immortalized porcine hepatocyte cell line, HepLi [40,41]. The study showed that PLGA nanoparticles coated with AS1411 were absorbed by QGY-7703 cells by the binding of nucleolin and that nanoparticles entered the cell by endocytosis and macro pinocytosis [40]. This study provides additional supporting evidence to show that AS1411 targets liver cancer cells by the recognition of aberrantly expressed nucleolin on the surface of cancer cells. However, this study did not assess the properties of the unconjugated AS1411 aptamer and did not study any of the potential cytotoxic effects of AS1411 *in vitro* or *in vivo*. Another recent study by Zhang and colleagues utilized mesoporous nanoparticles conjugated with Dox and AS1411 and cytochrome *c*, this study assessed the efficacy of these novel conjugates *in vitro* and *in vivo* using Hep-G2 cells [42]. The study demonstrated that nanoparticles conjugated with Dox and AS1411 could reduce tumor growth *in vivo* while preventing associated weight loss in treated mice, thus also supporting AS1411 as a liver cancer specific drug. However, the study did not address concerns regarding the cardio cytotoxicity and renal cytotoxicity of nanoparticle conjugate with doxorubicin, which is an important aim in such a study. In addition, the study demonstrated the accumulation of nanoparticles in the liver, spleen and lung without assessing any putative side effects or potential toxicity, which may be caused by nanoparticle accumulation. This is an important issue, as silica based nanoparticles have been shown to induce an increase in the fraction of insoluble ubiquitinated proteins in the tissues that they accumulate in. Accumulation of these nanoparticles has been linked to accelerated ageing in *c*.*elegans* [43].

## Conclusions

We have developed a novel adduct, which was prepared by the inexpensive and simple method of crosslinking the aptamer with doxorubicin using formaldehyde as a crosslinking agent. The dox-adduct has a similar binding affinity towards Huh7 cells when compared with the original aptamer, as determined by the dissociation constant of AS1411-Dox when compared with the AS1411 aptamer. We also examined the protective effect of conjugating Dox with AS1411. Additionally we examined the utility of the aptamer AS1411 as a stain for liver tumor histology, for the detection of plasma membrane-bound nucleolin in clinical liver tumor tissues. Here we show that AS1411 did not induce apoptosis in Huh7 cells, but instead induced cell cycle arrest in Huh7 cells at the G2/M checkpoint, in both time and dose dependent manners. Previous attempts to ameliorate the non-target side effects of anthracycline based treatments have involved the conjugation of anthracyclines with other drugs, in order to reduce cytotoxicity [30, 31, 44, 45]. However this approach does not address the issue of untargeted drug delivery. We also show that the AS1411-Dox adduct is able to efficiently deliver Dox into Huh7 cells with the same efficiency as free doxorubicin; this shows that conjugation with the aptamer does not prevent the drug from being released into cancer cells. In addition, *in vivo* testing of the AS1411-Dox adduct indicated that this drug-DNA adduct is well tolerated with few side effects. AS1411-Dox inhibits Huh7 tumor progression as measured by a reduction in tumor volume with almost the same efficiency as free doxorubicin but without the severe side effects. Total body weight loss was found to be significantly lower in mice treated with AS1411-Dox, compared with mice treated with Dox alone. This is important as many cancer patients experience severe weight loss during chemotherapy due to poor drug tolerance. Other serious side effects of doxorubicin include; cardio toxicity which can lead to heart failure and nephro-cytotoxicity that can eventually lead to renal failure. We showed by *in vivo* studies that AS1411-Dox is well tolerated compared with free Dox. Mice treated with either doxorubicin or AS1411-Dox or control-Dox all showed evidence of Caspase 3 dependent apoptosis in tumor cells. However, mice treated with either of the Dox adducts did not show evidence of Caspase 3 dependent apoptosis in either heart or kidney tissues. This shows that DNA-Dox adducts are protective against non-target tissue cytotoxicity, as both AS1411 and control DNA adducts did not show evidence of apoptosis in non-target tissues. Currently doxorubicin is administered as a course of chemotherapy for liver cancer patients with unresectable cancer as a palliative treatment. Doxorubicin is toxic to normal cells and is therefore administered intra-hepatically as either a drug emulsified in lipidodal (DLIP) or as a drug loaded into drug eluting beads (DEB) [46–49]. However, both are relatively inefficient methods of delivery; DLIP releases Dox very rapidly and is linked to tissue cardio toxicity and DEB releases Dox slowly which limits the efficacy of Dox on the tumor [39,46–49]. The high specificity of aptamer-mediated targeting with the combination of drug adducts, has the potential to replace such delivery methods such as DLIP and DEB [50,51]. AS1411-Dox could be a viable alternative for the administration of Dox for these liver cancer patients, as the non-specific cytotoxic effects (as analyzed so far) are minimal and the aptamer is highly specific for liver cancer. This adduct could also be amenable for the treatment of other cancers which highly express nucleolin. Especially in renal cell carcinoma where some efficacy with the AS1411 aptamer is reported [16]. Overall, the high specificity to cancer and the resultant reduced side effects of AS1411-Dox make it an extremely attractive candidate for further testing and potentially clinical trial, especially in use for patients with unresectable tumors.

## Supporting Information

S1 Fig AS1411 and nucleolin antibody staining of both Huh7 and U87 tumor cells. Both Huh7 and U87 tumor cells were stained with the nuclear stain DAPI (blue) and with either AS1411-FITC or the nucleolin antibody labeled with FITC. Both Huh7 and U87 cells showed strong membrane staining of nucleolin by AS1411 but not by the nucleolin antibody.
(TIF)

S2 Fig AS1411 and nucleolin antibody staining of both Huh7 and U87 liver tumor tissue. Hepatocellular carcinoma tissue slides were stained with biotinylated AS1411 or biotinylated nucleolin antibody and the intensity of staining was observed under light microscope. Images were first observed under 20x power and then sections were zoomed in at 40 x power. The tissue stains show strong binding by AS1411 compared with that of the nucleolin antibody.
(TIF)

S3 Fig HPLC chromatograph displaying the purification of AS1411-Dox adduct from reaction mixture. Showing DNA-Drug adduct at 260 nm and free doxorubicin at 490 nm.
(TIF)

S4 Fig Structure of doxorubicin bound to double stranded DNA. Doxorubicin (shown in blue) forms methylene links (dotted lines) via formaldehyde with N2 on both DNA strands (shown in green). Adapted from Zeman *et al* 1998.
(TIF)

S1 Table Sequences of DNA probes. Oligonucleotide probes for AS1411 and the control Aptamer are shown. Biotin, if applicable, was labelled at the 5'-end of probes.
(DOCX)
