# Supplementary figures and images for: A Synthetic Aptamer-Drug Adduct for Targeted Liver Cancer Therapy
Source: PLoS One. 2015 Nov 2;10(11):e0136673. doi: 10.1371/journal.pone.0136673 (PMC4629891; doi:10.1371/journal.pone.0136673)

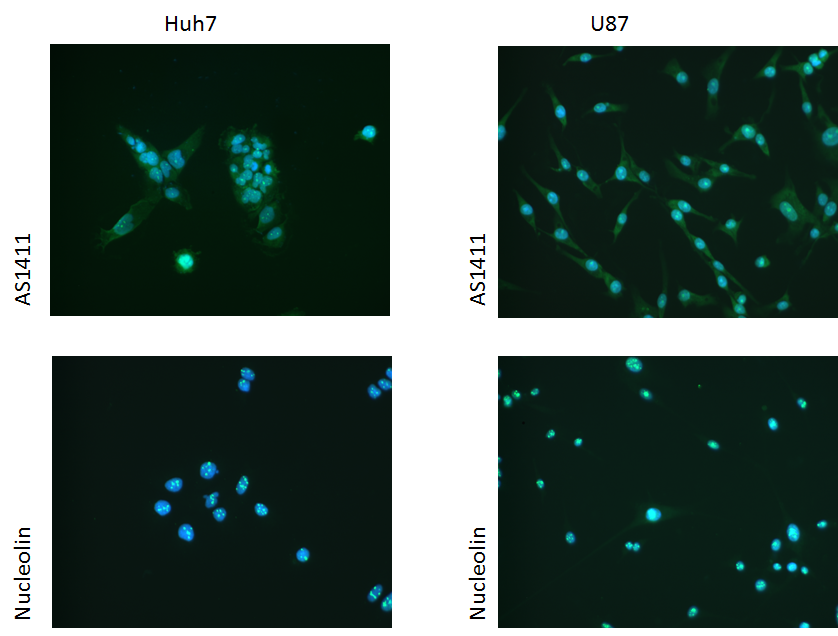

Supplement: S1 Fig — Both Huh7 and U87 tumor cells were stained with the nuclear stain DAPI (blue) and with either AS1411-FITC or the nucleolin antibody labeled with FITC. Both Huh7 and U87 cells showed strong membrane staining of nucleolin by AS1411 but not by the nucleolin antibody. (TIF) [file pone.0136673.s001.tif]

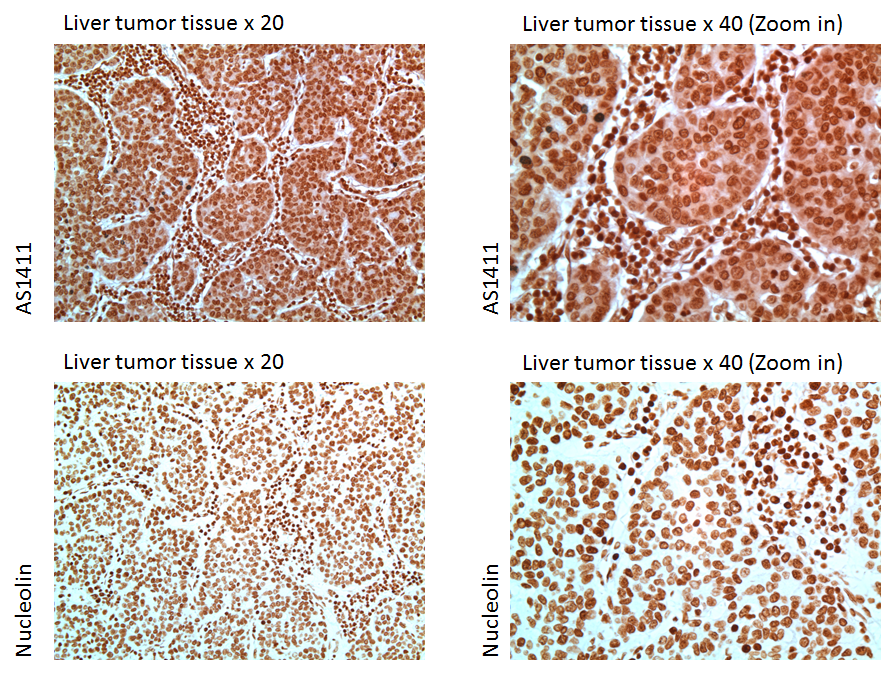

Supplement: S2 Fig — Hepatocellular carcinoma tissue slides were stained with biotinylated AS1411 or biotinylated nucleolin antibody and the intensity of staining was observed under light microscope. Images were first observed under 20x power and then sections were zoomed in at 40 x power. The tissue stains show strong binding by AS1411 compared with that of the nucleolin antibody. (TIF) [file pone.0136673.s002.tif]

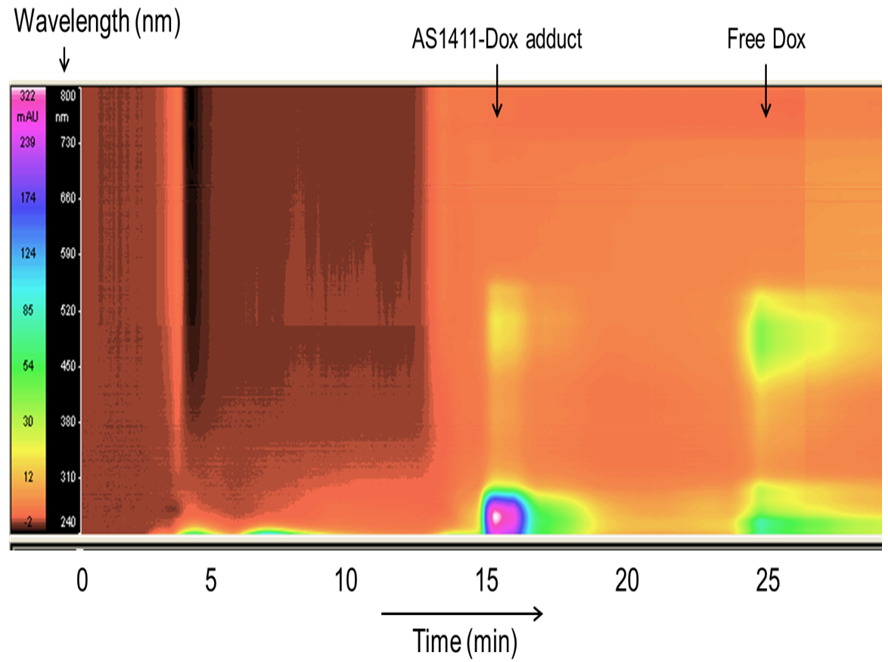

Supplement: S3 Fig — Showing DNA-Drug adduct at 260 nm and free doxorubicin at 490 nm. (TIF) [file pone.0136673.s003.tif]

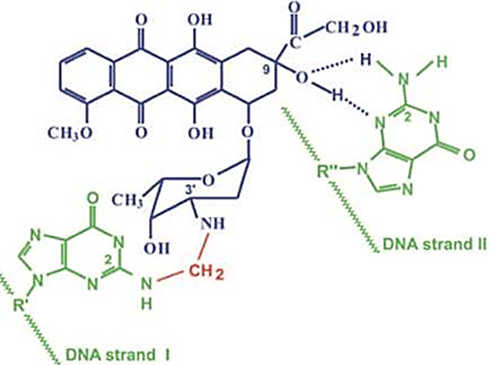

Supplement: S4 Fig — Doxorubicin (shown in blue) forms methylene links (dotted lines) via formaldehyde with N2 on both DNA strands (shown in green). Adapted from Zeman et al 1998. (TIF) [file pone.0136673.s004.tif]
