# Supplementary material for: A Synthetic Aptamer-Drug Adduct for Targeted Liver Cancer Therapy
Source: PLoS One. 2015 Nov 2;10(11):e0136673. doi: 10.1371/journal.pone.0136673 (PMC4629891; doi:10.1371/journal.pone.0136673)
Supplement: S1 Table — Oligonucleotide probes for AS1411 and the control Aptamer are shown. Biotin, if applicable, was labelled at the 5'-end of probes. (DOCX) [file pone.0136673.s005.docx]

| ***DNA*** | ***Sequences (5***'***-3***'***)*** |
| --- | --- |
| **AS1411** | GGT GGT GGT GGT TGT GGT GGT GGT GG |
| **Control DNA** | CGA CGA CGA CGA CGA CGA CGA CGA CGA CGA |
